# VIRTUAL REALITY FOR PALLIATIVE CARE COMMUNICATION TRAINING: A NOVEL DISSEMINATION STRATEGY

**DOI:** 10.1093/geroni/igad104.1083

**Published:** 2023-12-21

**Authors:** Kei Ouchi

**Affiliations:** Brigham and Women’s Hospital/Harvard Medical School, Boston, Massachusetts, United States

## Abstract

Towards the end of life, 75% of older adults visit the emergency department (ED). During acute health decompensation, most older adults with serious illness (predicted survival of less than one year) do not have advance directives and are at risk of receiving care inconsistent with their goals. To deliver patient-centered care, palliative care communication skills are essential, especially during the shared decision-making for cardiopulmonary resuscitation (i.e., code status conversations). Facilitated by skilled faculty and trained actors playing the role of a seriously ill older adult in acute health decompensation, the traditional palliative care communication training combines role play and individual feedback to practice communication skills. These evidence-based, palliative care trainings have demonstrated to improve communication skills in trained clinicians with enduring skills 12-15 months later. However, these trainings take a minimum of four hours with highly trained faculty and professional actors to role play, which hinders wider dissemination. To improve nationwide dissemination of evidence-based practices in code status conversations, we have leveraged virtual reality technology to allow clinicians to experience in-person, palliative care communication training. In collaboration with a virtual reality company, a 360-degree view, virtual reality training content has been filmed in a real hospital environment. Using the same evidence-based, palliative care communication contents, clinicians in training can now experience the role play in a virtual reality environment in one hour instead of four. By leveraging virtual reality technology, evidence-based palliative care communication training can now be disseminated with much less institutional investment (e.g., training time, faculty resources, etc.).

